# EHMT2 Inhibition Induces Cell Death in Human Non-Small Cell Lung Cancer by Altering the Cholesterol Biosynthesis Pathway

**DOI:** 10.3390/ijms21031002

**Published:** 2020-02-03

**Authors:** Haeun Kim, Seo Yoon Choi, Jinyeong Lim, Anders M. Lindroth, Yoon Jung Park

**Affiliations:** 1 Department of Nutritional Science and Food Management, Ewha Womans University, Seoul 03760, Korea; 2 Graduate School of Cancer Science and Policy, Cancer Biomedical Science, National Cancer Center, Goyang-si 10408, Korea

**Keywords:** EHMT2, epigenetics, cancer metabolism, cholesterol synthesis, lung cancer, autophagy

## Abstract

Non-small cell lung cancer (NSCLC) is a major subtype of lung cancer. Besides genetic and environmental factors, epigenetic alterations contribute to the tumorigenesis of NSCLC. Epigenetic changes are considered key drivers of cancer initiation and progression, and altered expression and activity of epigenetic modifiers reshape the epigenetic landscape in cancer cells. Euchromatic histone-lysine N-methyltransferase 2 (EHMT2) is a histone methyltransferase and catalyzes mono- and di-methylation at histone H3 lysine 9 (H3K9me1 and H3K9me2, respectively), leading to gene silencing. EHMT2 overexpression has been reported in various types of cancer, including ovarian cancer and neuroblastoma, in relation to cell proliferation and metastasis. However, its role in NSCLC is not fully understood. In this study, we showed that EHMT2 gene expression was higher in NSCLC than normal lung tissue based on publicly available data. Inhibition of EHMT2 by BIX01294 (BIX) reduced cell viability of NSCLC cell lines via induction of autophagy. Through RNA sequencing analysis, we found that EHMT2 inhibition significantly affected the cholesterol biosynthesis pathway. BIX treatment directly induced the expression of *SREBF2*, which is a master regulator of cholesterol biosynthesis, by lowering H3K9me1 and H3K9me2 at the promoter. Treatment of a cholesterol biosynthesis inhibitor, 25-hydroxycholesterol (25-HC), partially recovered BIX-induced cell death by attenuating autophagy. Our data demonstrated that EHMT2 inhibition effectively induced cell death in NSCLC cells through altering cholesterol metabolism-dependent autophagy.

## 1. Introduction

Lung cancer is globally the leading cause of cancer death according to World Health Organization (WHO) [1]. Non-small cell lung cancer (NSCLC), a major subtype of lung cancer, accounts for about 85% of total diagnosed lung cancers [2]. Diverse risk factors, including smoking, exposure to asbestos and radon, and genetic mutations have been well identified. Recently, epigenetic alteration has been highlighted as a driving force of tumorigenesis [3,4]. Epigenetic changes are considered a hallmark of cancer and a key driver of cancer initiation and progression [5,6]. Aberrant expression and activity of epigenetic modifying enzymes change the epigenetic landscape in the cancer cell.

Euchromatic histone-lysine N-methyltransferase 2 (EHMT2), also known as G9A, is a nuclear histone methyltransferase which mainly catalyzes mono- and di-methylation at histone H3 lysine 9 (H3K9me1 and H3K9me2, respectively). Increased H3K9me1 and H3K9me2 are generally associated with gene silencing in euchromatin [7]. Overexpression of EHMT2 has been reported in various cancers including ovarian cancer and lung cancer [8,9]. In lung cancer, overexpressed EHMT2 was reported to contribute to rapid proliferation and invasion [10,11], suggesting that it can be a potential therapeutic target. Nevertheless, the underlying mechanism behind EHMT2 overexpression is not fully understood.

One possible mechanism depends on a link between EHMT2 and cell metabolism. A previous study has reported that EHMT2 promoted proliferation of neuroblastoma by modulating cellular amino acid metabolism [12], suggesting a role of EHMT2 in cancer metabolism. The importance of metabolism in cancer cells has been recently revisited and emphasized [13]. Highly proliferative cancer cells tend to have limited levels of nutrient and oxygen availability [14,15], promoting nutrient uptake to support energetic and biosynthetic pathways and facilitating absorption of macromolecules [16]. Among macromolecule metabolisms, alterations of lipid metabolism have been reported in cancers of the breast, prostate, lung, and colon [17] and contribute to growth, energy and redox homeostasis, and metastases (reviewed in [18]). In particular, cholesterol has important roles in the composition of cellular membranes, hormone synthesis, and signal transduction pathways [19]. Upregulation of its biosynthesis has been reported in cancer [20] and gene expression involved in the biosynthesis is correlated with patient survival in specific types of cancer [21]. Fatty-acid and cholesterol biosynthesis is regulated by master regulators such as sterol regulatory element-binding proteins (SREBPs) [22]. There are three isoforms: SREBP1a, SREBP1c, and SREBP2. SREBP1a mainly controls fatty acid, phospholipid, and triacylglycerol synthesis, while SREBP2 does cholesterol synthesis [22]. SREBP2, encoded by the *SREBF2* gene, has been demonstrated to support cell survival in prostate cancer through accumulation of cholesterol, and its inhibition is suggested as a potential cancer therapy [23].

In this study, we investigated a novel link between epigenetic alteration and cancer metabolism, targeting NSCLC. We found that inhibition of EHMT2 activity induced cell death through autophagy and the cell death was mediated by activating cholesterol biosynthesis pathway. Our data suggest that epigenetic control of EHMT2 could be an important regulator of cancer metabolism in NSCLC cells.

## 2. Results

### 2.1. Overexpression of EHMT2 in NSCLC

**Figure 1:**
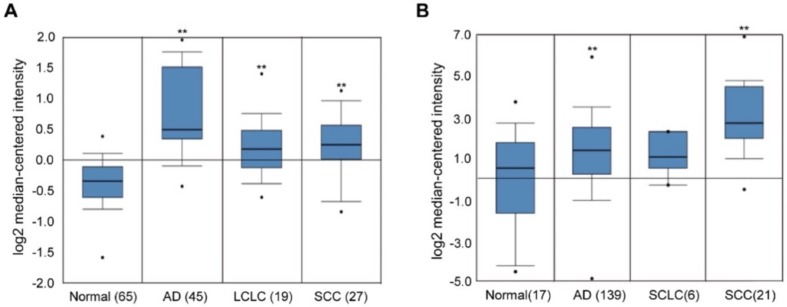
Euchromatic histone-lysine N-methyltransferase 2 (EHMT2) expression in different types of lung cancer. (**A** and **B**) Expression of lung cancer datasets for the *EHMT2* gene was presented using the Oncomine database. The data were extracted from the Hou lung dataset (**A**) and the Bhattacharjee lung dataset (**B**). *EHMT2* expression in different types of lung cancers was shown in a number of samples. AD: lung adenocarcinoma, LCLC: large cell lung carcinoma, SCC: squamous cell lung carcinoma, SCLC: small cell lung carcinoma. * *p* < 0.05, ** *p* < 0.001 against the normal tissues by *t*-test.

To examine the expression levels of *EHMT2* in different types of lung cancers, two datasets publicly available from Oncomine database (http://www.oncomine.com/) [24] were analyzed (Figure 1A,B): the Hou lung data set [25] and the Bhattacharjee lung dataset [26]. *EHMT2* expression was significantly higher in NSCLC, including adenocarcinoma (AD), squamous cell carcinoma (SCC), and large cell lung cancer (LCLC), compared to normal tissue, while it did not show a significant difference in small cell lung cancer (SCLC). On the contrary, *EHMT1*, another lysine methyltransferase, did not show differential expression in NSCLC (data not shown). In addition, high *EHMT2* protein expression was significantly correlated with poor prognosis (Supplementary Figure S1). Together, this suggests that *EHMT2* overexpression is a relevant cancer characteristic with possible ties to tumorigenesis.

### 2.2. Effects of EHMT2 Regulation on Cell Viability

**Figure 2:**
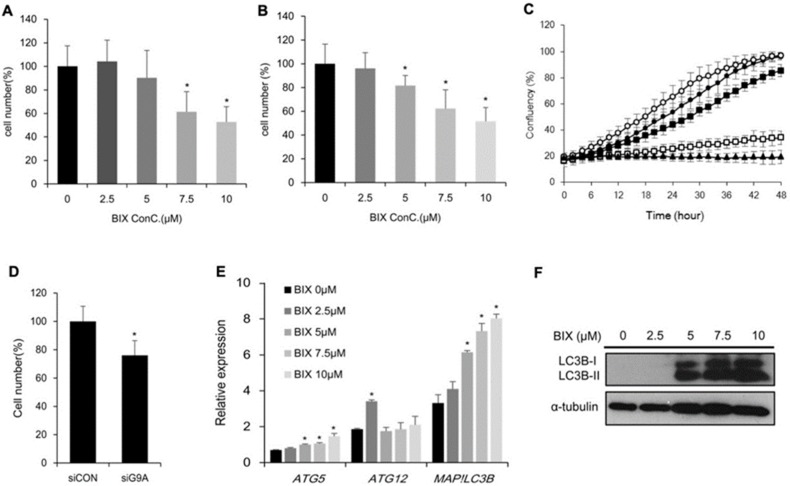
Suppression of cell proliferation and induction of autophagy by EHMT2 inhibition. (**A** and **B**) MTT assay of H1299 (**A**) and A549 (**B**) groups treated with BIX01294 (BIX) for 48 h was presented relative to the non-treated group. *p < 0.05 versus BIX non-treated group. (**C**) Cell confluency was measured by the IncuCyte Zoom live-imaging system in BIX-treated H1299. (**D**) MTT assays of H1299 cells were conducted after transfection with siCON or small interfering RNA targeting EHMT2 (siEHMT2) for 48 h. *p < 0.05, siCON versus siEHMT2 group. (**E**) Expression of the autophagy-related genes *ATG5*, *ATG12*, and *MAP1LC3B* was measured in BIX treated H1299. * *p* < 0.05 against 0 μM BIX treatment. (**F**) LC3B protein levels were analyzed by Western blotting after BIX treatment for 48 h in H1299. α-Tubulin levels are shown as loading control.

Next, we investigated the effects of EHMT2 in lung cancer cell lines H1299 and A549 to understand the role of EHMT2 in cancer cell proliferation. The cells were treated with the EHMT2-specific inhibitor BIX01294 (BIX), and cell viability was measured using the MTT assay. Cell viability decreased after BIX treatment as compared to non-treatment in both cell lines (Figure 2A,B). Also, we examined cell growth in the IncuCyte Zoom system and found that EHMT2 inhibition by BIX treatment hindered cell proliferation in a dose-dependent manner (Figure 2C). Since 7.5 μM of BIX treatment was the minimal concentration that showed a significant difference in cell viability, this concentration was used for further experiments. In addition, transcriptional repression of *EHMT2* mediated by specific targeting siRNA significantly decreased cell viability (Figure 2D). To elucidate the mechanism of suppressing cell proliferation by EHMT2 inhibition, we tested whether BIX-induced cell death was mediated by autophagy. The autophagy-related genes *Autophagy Related gene 5 (ATG5), Autophagy Related gene 12 (ATG12),* and *Microtubule-associated proteins 1A/1B light chain 3B (MAP1LC3B)*, were measured upon BIX-treatment. The mRNA level of *ATG5* and *MAP1LC3B*, not *ATG12*, significantly increased in a dose-dependent manner (Figure 2E). Western blot analysis using an antibody against LC3B, encoded by the *MAP1LC3B* gene, confirmed the autophagy induction by BIX-treatment (Figure 2F). These results suggested that EHMT2 inhibition induced cell death through autophagy.

### 2.3. Distinct Gene Expression Profiles with EHMT2 Inhibition in H1299 Cells

**Figure 3:**
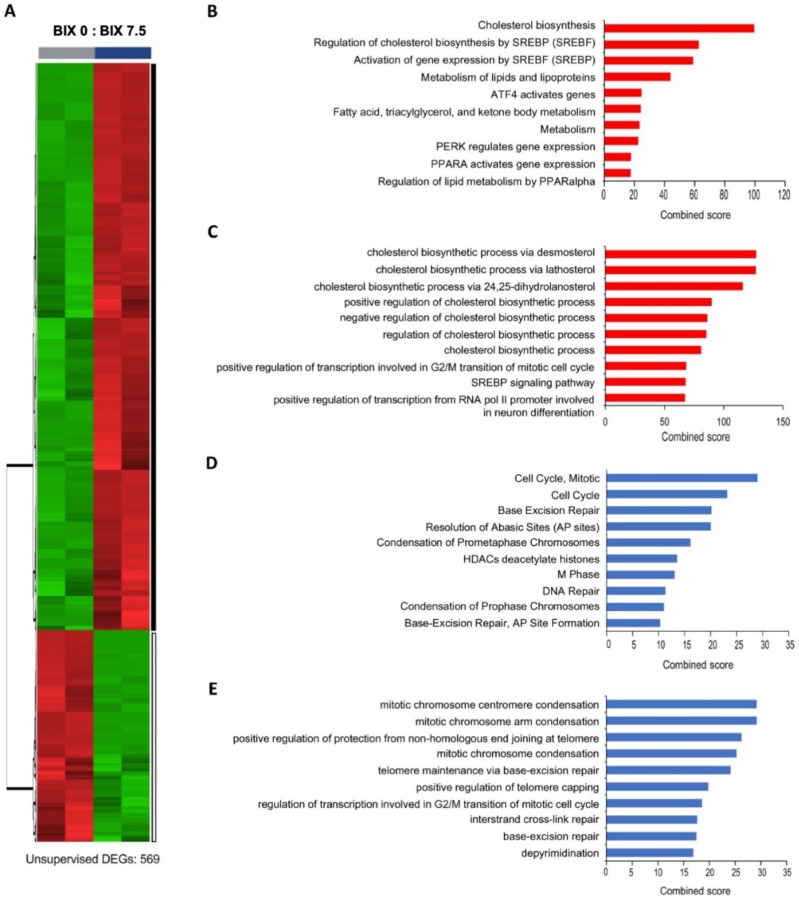
Distinct expression profiles in the BIX-treated H1299 cell line using RNA sequencing. (**A**) The gene expression pattern according to RNA sequencing in heatmap. Black bar: 422 upregulated differentially expressed genes (DEGs). White bar: 147 downregulated DEGs. (**B**) Top 10 Reactome pathways of upregulated genes. (**C**) Top 10 Gene Ontology (GO) biological processes (BPs) terms of upregulated genes. (**D**) Top 10 Reactome pathways of downregulated genes. (**E**) Top 10 GO BP terms of downregulated genes based on combined score obtained from Enrichr.

To understand the effects of EHMT2 inhibition on global gene expression, RNA sequencing analysis was conducted on cells treated with or without BIX. EHMT2 inhibition exhibited distinct gene expression profiles (Figure 3A). In total, 569 genes out of 23,912 genes passed the cutoff (*p* value < 0.05 and log_2_FC ≥ |0.6|) and among them, 147 genes (26%) were downregulated and 422 genes (74%) were upregulated. The major biological function of differentially expressed genes (DEGs) was analyzed by biological process (BP) of Gene Ontology (GO) and Reactome using Enrichr. Interestingly, metabolism-related terms were overrepresented in upregulated DEGs, where the cholesterol biosynthesis pathway was a top ranked biological term from both the Reactome and GO BP pathways (Figure 3B,C). The majority of downregulated DEGs were, in contrast, involved in cell cycle-related processes, including mitotic chromosome condensation and DNA repair pathways (Figure 3D,E). Taken together, BIX-mediated EHMT2 inhibition induced the cholesterol biosynthesis pathway and repressed cell cycle and DNA repair pathways.

### 2.4. Induction of Cholesterol Biosynthesis Pathway by BIX Treatment

**Figure 4:**
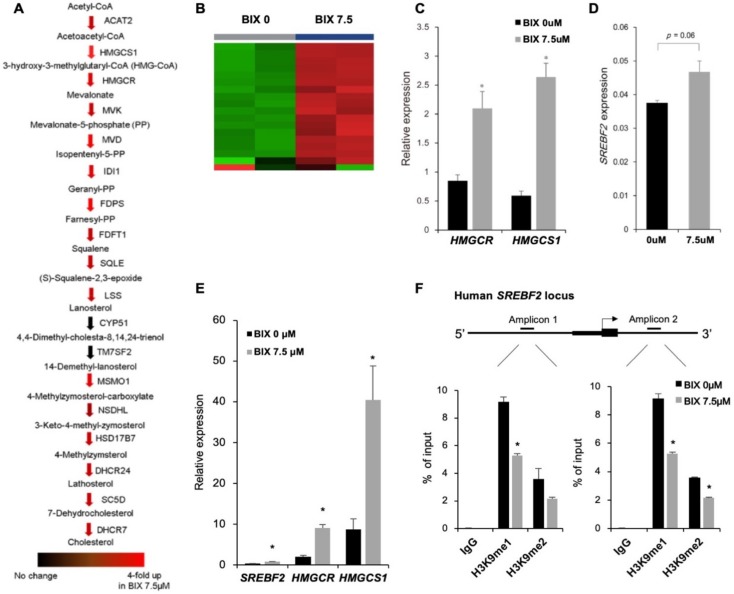
Induction of the cholesterol biosynthesis pathway by direct regulation of BIX in the *SREBF2* locus. (**A**) Expressions of genes, annotated as cholesterol biosynthesis, were presented with colored arrows, based on RNA sequencing data. A range of redness indicates a level of upregulation in the 7.5 μM BIX-treated group, as compared to non-treated group. (**B**) The expression pattern of cholesterol synthesis-related genes shown in heatmap according to RNA sequencing of H1299 with non-treatment or 7.5 μM of BIX treatment. (**C**) Expression of genes in cholesterol biosynthesis was validated by qRT-PCR of *HMGCR* and *HMGCS1*. (**D**) Expression of *SREPF2* was validated by qRT-PCR. (**E**) Expression of cholesterol synthesis-related genes in BIX-treated A549 was analyzed. (**F**) Results from chromatin immunoprecipitation using antibodies against histone H3 lysine 9 mono-methylation (H3K9me1) and histone H3 lysine 9 di-methylation (H3K9me2) were presented as % enrichment of input at the promoter of *SREBF2*. IgG is a negative control for immunoprecipitation. * *p* < 0.05 against the 0 μM BIX treatment group.

Next, we focused on cholesterol biosynthesis pathway, which was identified as a top candidate of BIX-induced transcriptional changes, according to Figure 3B,C. We checked expression of individual genes related to cholesterol biosynthesis pathway (Figure 4A,B). Most of cholesterol biosynthesis related genes, except *CYP51* and *TM7SF2,* were significantly upregulated by EHMT2 inhibition, indicating that BIX-treatment promoted overall cholesterol biosynthesis pathway. Moreover, the expression of *HMGCR* and *HMGCS1*, which are the rate-limiting step of cholesterol biosynthesis [27], was verified by qRT-PCR. Both mRNA level of *HMGCR* and *HMGCS1* increased significantly after BIX treatment (Figure 4C).

To identify how integral cholesterol biosynthesis was regulated by BIX-treatment, we investigated the alteration of the cholesterol biosynthesis master regulator, SREBF2. We found that *SREBF2* expression significantly increased upon EHMT2 inhibition (Figure 4D). In addition, expression of *SREBF2*, along with *HMGCR* and *HMGCS1*, was also increased after BIX treatment in A549 (Figure 4E). To further characterize *SREBF2* regulation by EHMT2, chromatin immunoprecipitation (ChIP) assay was performed with H3K9me1 and H3K9me2 antibodies. Inhibition of EHMT2 significantly reduced the enrichment of H3K9me1 and H3K9me2 at the promoter of the *SREBF2* locus (Figure 4F). This suggested that EHMT2 inhibition directly upregulated *SREBF2* transcription by lowering the enriched level of H3K9me1 and H3K9me2, leading to induction of cholesterol biosynthesis.

### 2.5. Involvement of Cholesterol Biosynthesis in BIX-Induced Cell Death

**Figure 5:**
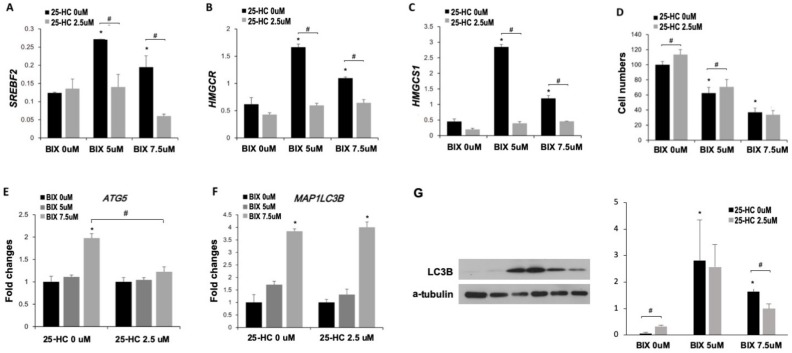
Involvement of cholesterol biosynthesis pathway in BIX-induced cell death. (**A**–**C**) Expression of *SREBF2* (**A**), *HMGCR* (**B**), and *HMGCS1* (**C**) was analyzed in BIX- and 25-hydroxycholesterol (25-HC)-treated H1299 cells. (**D**) MTT assay was conducted in lysates of H1299 with BIX and 25-HC treatment, based on 0 μM of BIX and 25-HC as 100%. (**E**,**F**) Expression of the autophagy-related genes *ATG5* (**E**) and *MAP1LC3B* (**F**) was analyzed in BIX- and 25-HC-treated H1299 cells. Data were normalized by the value of 0 μM BIX in each 25-HC treatment. (**G**) LC3B protein levels were analyzed by Western blotting after BIX and 25-HC treatment for 48 h in H1299 cells. α-Tubulin levels are shown as loading control. Relative intensities of LC3B-II bands against a-tublin were quantified and presented in the right panel. * *p* < 0.05 versus 0 μM BIX treatment. # *p* < 0.05 versus 2.5 μM 25-HC treatment.

To confirm that cholesterol biosynthesis was involved in BIX-induced cell death, cells were co-treated with BIX and 25-hydroxycholesterol (25-HC), an inhibitor of cholesterol biosynthesis [28]. BIX increased the expression of *SREBF2*, *HMGCR,* and *HMGCS1*, while in contrast, their expressions were significantly reduced by the 25-HC treatment (Figure 5A–C). Interestingly, 25-HC did not change the expression of genes related to cholesterol biosynthesis pathway in the absence of BIX treatment (Figure 5A–C), suggesting that the induction of cholesterol biosynthesis pathway occurred upstream in response to BIX treatment. Based on the MTT assay, cell viability decreased upon EHMT2 inhibition, but 25-HC treatment recovered the decreased cell viability, at least in part (about 13%) (Figure 5D). Since we discovered that BIX-induced cell death was involved in autophagy, we hypothesized that cholesterol biosynthesis pathway may be related to autophagy. Thus, we investigated whether inhibition of cholesterol synthesis pathway attenuated the BIX-induced autophagy. The expression of autophagy-related genes was measured at mRNA and protein level. We found that expression of *ATG5* and *MAP1LC3B* was significantly upregulated by EHMT2 inhibition, and the upregulation was attenuated by 25-HC treatment at the mRNA level of *ATG5*, but not *MAP1LC3B* (Figure 5E,F). Furthermore, 25-HC treatment also decreased the level of the LC3B protein upon EHMT2 inhibition, although the basal level in the absence of BIX was higher in 25-HC-treated group. The results indicated that cholesterol biosynthesis pathway was involved in BIX-induced cell death via autophagy.

## 3. Discussion

In this study, we showed that the inhibition of the histone methyltransferase EHMT2 using BIX induced cell death via autophagy. Most cholesterol biosynthesis-related genes were upregulated upon BIX-treatment. The link between EHMT2 and cholesterol biosynthesis was based on the finding that EHMT2 inhibition led to reduced H3K9me1 and H3K9me2 at the promoter of the *SREBF2* locus, resulting in transcriptional upregulation of *SREBF2* and its target genes. The 25-HC-mediated inhibition of cholesterol biosynthesis recovered BIX-induced cell death via attenuating autophagy. Therefore, inhibition of EHMT2 could be a potential approach to induce cell death via alteration of expression of *SREBF2* and its downstream cholesterol synthesis.

NSCLC has higher EHMT2 expression compared to the normal tissues according to the Oncomine datasets (Figure 1). The anti-cancer effect of EHMT2 inhibition has been studied in several cancer cell lines in primary cells of breast cancer, neuroblastoma, colon, and bladder cancer [12,29,30], suggesting possible therapeutic options for cancer treatment. Our results showed that EHMT2 inhibition led to cell death in NSCLC cells, associated with BIX-mediated induction of autophagy. Autophagy has been demonstrated to contribute to cell death in the absence of the apoptotic pathway [31]. ATG5 is constitutively conjugated to ATG12 and forms the ATG5-ATG12 complex, which is necessary for LC3B lipidation [32]. This process is required for autophagosome formation [33]. BIX treatment resulted in transcriptional induction of *MAP1LC3B* and *ATG5* genes and an increase of LC3B-II production. Consistent with our result, previous reports also showed that EHMT2 inhibition induced autophagic cell death since suppression of autophagy by an inhibitor or siRNA knockdown reduced BIX-mediated cell death in other cell types including breast, pancreatic, and colon cancer cell lines [30,34,35]. In addition, we also observed apoptotic cell death upon the treatment although the extent was limited (Supplementary Figure S2).

Genome-wide RNA sequencing upon BIX-treatment revealed that the most prominent change in gene expression was induction of metabolic pathways, in particular, cholesterol biosynthesis (Figure 2B,C). There have been a few studies about the roles of EHMT2 and its association with metabolism [12]. EHMT2 epigenetically activates the serine-glycine biosynthetic pathway, which is critical for cancer cell survival and proliferation through ribosome synthesis and cell cycle progression [12]. It also influences insulin regulation in hepatic cells by activating *HMGA1* independently of its enzymatic activity [36]. Based on our findings, EHMT2 seems to directly regulate transcriptional expression of *SREBF2,* a master regulator for cholesterol biosynthesis, in effect lowering cholesterol in lung cancer. We proved this experimentally by blocking EHMT2, derepressing *SREBF2* resulting from decreased H3K9me1 and H3K9me2, leading to transcriptional induction of canonical downstream target genes. Similar to our results, it has been reported that BIX treatment selectively induced upregulation of cholesterol biosynthesis in pancreatic cancer cells [35]. However, the BIX-induced upregulation did not take place in the liver cancer cells [35], indicating that it is cell-type specific.

Moreover, an SREBP2-mediated induction of the cholesterol biosynthesis pathway was involved in cell death in part through the induction of autophagy. We also proved this experimentally by showing that 5-HC-mediated inhibition of cholesterol synthesis attenuated expression of *ATG5* (Figure 4E). While it did not influence *MAP1LC3B* mRNA expression, it did reduce LC3B-II, likely as a result of the absence of ATG5, a key component of the LC3B-II binary complex. To corroborate our findings, previous studies have shown the relationship between cholesterol synthesis or SREBP2 and autophagy [37,38]. SREBP2 directly binds to the promoter of several murine autophagy genes, such as *Map1lc3b, Atb4b,* and *Atg4d*, and SREBF2 knockout disrupts autophagosome formation in nutrient starvation in mouse models [37]. Gastric cancer cells also represent a case where cholesterol supplement in the culture media decreased cell viability and clonogenicity via both autophagy and apoptosis [38]. In our results, it is not clear whether autophagy was induced by direct activation via SREBF2 binding to the promoters of autophagy genes or by cholesterol supplement for autophagosome formation. There have also been conflicting results suggesting cholesterol depletion-induced autophagy [39] and an inverse link between cholesterol biosynthesis and high cell proliferation rate in other cancers [40]. Further investigation is needed to elucidate why EHMT2 inhibition-induced autophagy required the cholesterol synthesis pathway. It nevertheless remains clear that cholesterol biosynthesis, EHMT2 transcriptional control, and autophagy are linked in ways that relate to proliferative activity and cell viability, likely essential to lung cancer-related tumorigenesis.

In conclusion, EHMT2 inhibition using the specific inhibitor BIX induced cell death via autophagy in NSCLC. It led to upregulation of cholesterol biosynthesis by derepressing *SREBF2* expression. EHMT2 inhibition-induced cell death was in part alleviated by suppressing the cholesterol biosynthesis pathway and consequently diminishing *ATG5* expression. A current model is demonstrated to show how EHMT2 inhibition influences cell viability via induction of cholesterol biosynthesis in NSCLC.

## 4. Materials and Methods

### 4.1. Cell Culture and Treatments

H1299 and A549 cell lines, obtained from the Korean Cell Line Bank, were maintained in RPMI1640 (Welgene, Daegu, Korea; LM011-51 and LM011-03, respectively) supplemented with 10% fetal bovine serum (Atlas, Fort Collins, CO, USA) and 2.5 μL/mL gentamicin (Thermo Fisher Scientific, Darmstadt, Germany; 15710-064). BIX01294 (BIX) (Sigma-Aldrich, Saint Louis, MO, USA; B9311) and 25-hydroxycholesterol (25-HC) (Sigma-Aldrich, Saint Louis, MO, USA; H1015) were dissolved in dimethyl sulfoxide (DMSO) (Sigma-Aldrich, Saint Louis, MO, USA; D4540) and ethanol (Millipore, Darmstadt, Germany; 64-17-5) at 24 mM and 10 mM for stock solutions, respectively.

### 4.2. Small Interfering RNA Transfection

For repression of target gene expression, cells were transfected with siRNAs targeting EHMT2 (siEHMT2) (Dharmacon, Lafatette, CO, USA; L-006937-00-0005) and scrambled, non-targeting control (siCON) (Dharmacon, Lafatette, CO, USA; D-001810-10-05). Cells were seeded at 0.3 × 10⁴ cells per well in 96-well plates, and the next day transfected with siEHMT2 or siCON (50 nmol/L) using DharmaFECT1 (Dharmacon; T-2001-01). Transfected cells were harvested after incubation for two days and used for further analysis.

### 4.3. MTT Assay for Cell Viability

Cells were seeded in 96-well plates 24 h prior to treatment. BIX were treated for 48 h. Thiazolyl blue tetrazolium bromide (MTT) (Sigma-Aldrich, Saint Louis, MO, USA; M2128) solution was added as one-tenth the original culture volume and incubated for 3 h. Media were removed and 100 μL DMSO (Sigma-Aldrich, Saint Louis, MO, USA; D4540) per well were added to convert MTT to purple formazan in mitochondria of viable cells. The optical density (OD) of each culture well was measured using an ELISA reader at 562 nm. The OD 562 in control cells was taken as 100%.

### 4.4. IncuCyte Zoom Assays

IncuCyte ZOOM System real-time instrumentation (Essen Bioscience, Ann Arbor, MI, USA) captures phase contrast images and measures cell proliferation. Cells were seeded in six-well plates and incubated for 24 h. After each treatment, plates were placed in IncuCyte. IncuCyte captured cell images every 2 h and the obtained images were analyzed by IncuCyte ZOOM™ 2015A software (Essen Bioscience, Ann Arbor, MI, USA).

### 4.5. RNA Isolation and Quantitative Reverse Transcription RT-PCR

Total RNA was isolated with Trizol (Ambion, Carlsbad, CA, UCA; 15596018) according to the manufacturer’s protocol. RevertAid Reverse Transcriptase (Thermo Fisher Scientific, Darmstadt, Germany) was used for cDNA synthesis. Quantitative reverse transcription PCR (qRT-PCR) was performed using the SYBR green PCR master mix (Qiagen, Hilden, Germany) by Rotor Gene Q (Qiagen, Hilden, Germany). All samples were normalized to *TATA-binding protein* (*TBP*) mRNA levels. Primers used for RT-PCR were: *ATG5* forward (5′-TGGAGTAGGTTTGGCTTTGG-3′), *ATG5* reverse (5′-ATGGTTCTG TTCCCTTTCA-3′), *ATG12* forward (5′-CCTTTGCTCCTTCCCCAGA-3′), *ATG12* reverse (5′-ATCCCCACGCCTGAGACTT-3′), *MAP1LC3B* forward (5′-GAGAGCAGCATCCAACCAAA -3′), *MAP1LC3B* reverse (5′-ACATGGTCAGGTACAAGGAAC-3′), *HMGCR* forward (5′-GTTTC AGTCCAGGTCAGGG-3′), *HMGCR* reverse (5′-GCAGCAGGTTTCTTGTCAGT-3′), *HMGCS1* forward (5′-CAGAAGAACTTACGCTCGGC-3′), *HMGCS1* reverse (5′- TCTTGGCAGGGCT GGAATA-3′), *SREBF2* forward (5′-GCAGAGTTCCTTCTGCCATT-3′), *SREBF2* reverse (5′-GCGACAGTAGCAGGTCACAG-3′), *TBP* forward (5′-AGCCAAGAGTGAAGAACAGTCC-3′) and *TBP* reverse (5′-CACAGCTCCCCACCATATTC-3′).

### 4.6. Chromatin Immunoprecipitation (ChIP) Analysis

Cells were seeded and after 24 h, the cells were treated with or without 7.5 μM BIX for 48 h. Cross-linking was performed with 1% formaldehyde by gentle shaking for 5 min and was quenched with 125 mM glycine for 5 min, both at room temperature. The cells were harvested and rinsed with PBS. The cell pellet was sonicated in a Covaris S220 focused ultrasonicator (Covaris, Woburn, MA) for nuclei isolation with the following settings: peak power 75 W, duty factor 2%, and 200 cycles/burst at 4 °C for 180 s. Proteinase inhibitors (PIs) at all stages were provided using cOmplete protease inhibitor cocktail (EDTA-free, Roche, Mannheim, Germany), 0.7 µg/mL pepstatin (Roche, Mannheim, Germany), and 1 µg/mL aprotinin (Roche, Mannheim, Germany). The sonicated cells were spun and were washed with nuclei isolation buffer (10 mM HEPES (pH 7.5), 85 mM KCl, 0.5% IGEPAL CA-630, and PIs). The isolated nuclei were resuspended with DNA shearing buffer (10 mM Tris-HCl (pH 8), 0.1% SDS, 1 mM EDTA, and PIs) and then were sonicated at the following settings: peak power 140 W, duty factor 2%, and 200 cycles/burst at 4 °C for 60 s for 48 cycles. After sonication, sheared DNA was diluted with ChIP-dilution buffer (0.01% SDS, 1.1% Triton X-100, 1.2 mM EDTA, 16.7 mM Tris-HCl, and 167 mM NaCl) and the chromatin was immunoprecipitated using ChIP grade anti-H3K9me1 (Abcam, Cambridge, UK; ab9045), H3K9me2 (Abcam, Cambridge, UK; ab1220), or control mouse IgG (Santa Cruz Biotechnology, Dallas, TX, USA; ab-2005). Each sample was analyzed by qPCR with primers covering the promoter of the *SREBF2* gene. Amplicons for the SREBF2 locus were designed using Encyclopedia of DNA Elements (ENCODE) [41,42]; amplicon 1 range from -2639 to -2528 and amplicon 2 range from +1099 to +1190. Primers are as follows: *SREBF2*_amplicon1 forward (5′-TGGGAGTTGTT GCTGAATCC-3′) and reverse (5′-ACAGCACTGA GCAGGAAGGT-3′), SREBF2_amplicon2 forward (5′-CCCAGCTGGTTAGAGCCTAGT-3′) and reverse (5′-GCCT AGTCAACTGGACTCTTTCTT-3′). 

### 4.7. Western Blot Analysis

Collected cell pellets were extracted in RIPA buffer (20 mM HEPES, pH 7.0, 150 mM NaCl, 10% glycerol, 1% Triton X-100, 1 mM EGTA, 10 mM β-Glycerophosphate, 1 mM Na_3_VO_4_, and 5 mM NaF) and protein concentrations were determined with the BCA protein assay kit (Thermo Fisher Scientific, Darmstadt, Germany; #23227). The proteins were separated on 12% gels by electrophoresis and were transferred to PVDF membranes (Merck Millipore, Billerica, MA, USA; ISEQ00010). The membranes were blocked in 0.5% BSA in Tris-buffered saline with Tween-20 (TBS-T) and were probed with: anti-LC3B (Cell signaling, Danvers, MA, USA; #3868) and anti-α-tubulin (Sigma-Aldrich, Saint Louis, MO, USA; T5168), followed by secondary HRP-antibodies; anti-mouse IgG (Santa Cruz Biotechnology, Dallas, TX, USA; sc-2005), and anti-rabbit IgG-HRP (Santa Cruz Biotechnology, Dallas, TX, USA; sc-2004). The blocking and probing were performed using SNAP i.d. 2.0 Protein Detection System (Merck Millipore, Billerica, MA, USA). Proteins were detected using Pierce ECL chemiluminescence kit (Thermo Fisher Scientific, Darmstadt, Germany; #32132). The bands were quantified by ImageJ (National Institutes of Health, Bethesda, MD, USA).

### 4.8. RNA-Sequencing Analysis

Total RNA from H1299, treated with or without BIX 7.5 μM, was isolated using RNeasy Mini Kit (Qiagen). RNA-seq was performed on the RNA with TrueSeq RNA kit for library preparation and paired-end sequencings on an Illumina HiSeq 2500 instrument by Macrogen Inc (Seoul, Korea). 5.8 Gbp read bases per sample on average were obtained with a Q30 phred quality score of 94–96%. The received data were processed with Tuxedo pipeline, resulting in average 31,573 peaks counted from cufflinks. Overlapped genes from data sheets of four samples were merged and removed unannotated genes. Finally, 23,912 peaks were selected and reads counts for each gene were converted into base 2 logarithm. For further analysis, the cutoff standard to display differentially expressed genes (DEGs) was set at *p*-value < 0.05 and log₂FC ≥ |0.6| (fold change 1.5), resulting in 569 genes. The DEGs were assessed by Gene Ontology (GO) biological process (BP) terms [43] and Reactome [44] using Enrichr, a comprehensive gene set enrichment analysis web server (http://amp.pharm.mssm.edu/Enrichr) [45].

### 4.9. Statistics

Statistical analysis was performed with Microsoft Excel and R studio using student’s *t*-test with two-tailed and equal variances. Data were visualized with means ± standard deviations.

## Supplementary Materials

Supplementary materials can be found at https://www.mdpi.com/1422-0067/21/3/1002/s1, Figure S1: Survival analysis in lung cancer depending on *EHMT2* expression; Figure S2: Apoptosis induced by BIX.

## Abbreviations

25-HC	25-hydroxycholesterol
AD	Adenocarcinoma
ATF4	Activating transcription factor 4
ATG	Autophagy-related gene
BIX	BIX01294
BP	Biological process
ChIP	Chromatin immunoprecipitation
DEG	Differentially expressed gene
DMSO	Dimethyl sulforxide
EHMT2	Euchromatic histone-lysine N-methyltransferase 2
FACS	Flow cytometry analysis
GO	Gene Ontology
H3K9	Histone H3 lysine 9
H3K9me1	Histone H3 lysine 9 mono-methylation
H3K9me2	Histone H3 lysine 9 di-methylation
HMGCR	HMG-CoA synthase 1
HMGCS1	HMG-CoA reductase
LCLC	Large cell carcinoma
MAP1LC3B	Microtubule-associated proteins 1A/1B light chain 3B
MTT	Thiazolyl blue tetrazolium bromide
NSCLC	Non-small cell lung cancer
qRT-PCR	Quantitative real-time PCR
SCC	Squamous cell cancer
SD	Standard deviation
siATF4	Small interfering RNA targeting ATF4
siCON	Non-targeting small interfering RNA
siEHMT2	Small interfering RNA targeting EHMT2
SREBF	Sterol regulatory element-binding transcription factor

## References

[1] World Health Organization, International Agency for Research on Cancer (2015). World Cancer Report 2014.

[2] Duma N., Santana-Davila R., Molina J.R. (2019). Non-Small Cell Lung Cancer: Epidemiology, Screening, Diagnosis and Treatment. Mayo Clin. Proc..

[3] Zochbauer-Muller S., Fong K.M., Virmani A.K., Geradts J., Gazdar A.F., Minna J.D. (2001). Aberrant promoter methylation of multiple genes in non-small cell lung cancers. Cancer Res..

[4] Sasaki H., Moriyama S., Nakashima Y., Kobayashi Y., Kiriyama M., Fukai I., Yamakawa Y., Fujii Y. (2004). Histone deacetylase 1 mRNA expression in lung cancer. Lung Cancer.

[5] Flavahan W.A., Gaskell E., Bernstein B.E. (2017). Epigenetic plasticity and the hallmarks of cancer. Science.

[6] Lindroth A.M., Park Y.J., Plass C., Meissner A., Walter J. (2015). Epigenetic Reprogramming in Cancer. Epigenetic Mechanisms in Cellular Reprogramming.

[7] Tachibana M., Sugimoto K., Fukushima T., Shinkai Y. (2001). Set domain-containing protein, G9a, is a novel lysine-preferring mammalian histone methyltransferase with hyperactivity and specific selectivity to lysines 9 and 27 of histone H3. J. Biol. Chem..

[8] Chen M.W., Hua K.T., Kao H.J., Chi C.C., Wei L.H., Johansson G., Shiah S.G., Chen P.S., Jeng Y.M., Cheng T.Y. (2010). H3K9 histone methyltransferase G9a promotes lung cancer invasion and metastasis by silencing the cell adhesion molecule Ep-CAM. Cancer Res..

[9] Hua K.T., Wang M.Y., Chen M.W., Wei L.H., Chen C.K., Ko C.H., Jeng Y.M., Sung P.L., Jan Y.H., Hsiao M. (2014). The H3K9 methyltransferase G9a is a marker of aggressive ovarian cancer that promotes peritoneal metastasis. Mol. Cancer.

[10] Huang T., Zhang P., Li W., Zhao T., Zhang Z., Chen S., Yang Y., Feng Y., Li F., Shirley Liu X. (2017). G9A promotes tumor cell growth and invasion by silencing CASP1 in non-small-cell lung cancer cells. Cell Death Dis..

[11] Cho H.S., Kelly J.D., Hayami S., Toyokawa G., Takawa M., Yoshimatsu M., Tsunoda T., Field H.I., Neal D.E., Ponder B.A. (2011). Enhanced expression of EHMT2 is involved in the proliferation of cancer cells through negative regulation of SIAH1. Neoplasia.

[12] Ding J., Li T., Wang X., Zhao E., Choi J.H., Yang L., Zha Y., Dong Z., Huang S., Asara J.M. (2013). The histone H3 methyltransferase G9A epigenetically activates the serine-glycine synthesis pathway to sustain cancer cell survival and proliferation. Cell Metab..

[13] Hanahan D., Weinberg R.A. (2011). Hallmarks of cancer: The next generation. Cell.

[14] Carmeliet P., Dor Y., Herbert J.M., Fukumura D., Brusselmans K., Dewerchin M., Neeman M., Bono F., Abramovitch R., Maxwell P. (1998). Role of HIF-1alpha in hypoxia-mediated apoptosis, cell proliferation and tumour angiogenesis. Nature.

[15] Izuishi K., Kato K., Ogura T., Kinoshita T., Esumi H. (2000). Remarkable Tolerance of Tumor Cells to Nutrient Deprivation: Possible New Biochemical Target for Cancer Therapy. Cancer Res..

[16] Boroughs L.K., DeBerardinis R.J. (2015). Metabolic pathways promoting cancer cell survival and growth. Nat. Cell Biol..

[17] Furuta E., Okuda H., Kobayashi A., Watabe K. (2010). Metabolic genes in cancer: Their roles in tumor progression and clinical implications. Biochim. Biophys. Acta.

[18] Santos C.R., Schulze A. (2012). Lipid metabolism in cancer. FEBS J..

[19] Maxfield F.R., van Meer G. (2010). Cholesterol, the central lipid of mammalian cells. Curr. Opin. Cell Biol..

[20] Larsson O. (1996). HMG-CoA reductase inhibitors: Role in normal and malignant cells. Crit. Rev. Oncol. Hematol..

[21] Kuzu O.F., Noory M.A., Robertson G.P. (2016). The Role of Cholesterol in Cancer. Cancer Res..

[22] Horton J.D. (2002). Sterol regulatory element-binding proteins: Transcriptional activators of lipid synthesis. Biochem. Soc. Trans..

[23] Lewis C.A., Brault C., Peck B., Bensaad K., Griffiths B., Mitter R., Chakravarty P., East P., Dankworth B., Alibhai D. (2015). SREBP maintains lipid biosynthesis and viability of cancer cells under lipid- and oxygen-deprived conditions and defines a gene signature associated with poor survival in glioblastoma multiforme. Oncogene.

[24] Rhodes D.R., Yu J., Shanker K., Deshpande N., Varambally R., Ghosh D., Barrette T., Pandey A., Chinnaiyan A.M. (2004). ONCOMINE: A cancer microarray database and integrated data-mining platform. Neoplasia.

[25] Hou J., Aerts J., den Hamer B., van Ijcken W., den Bakker M., Riegman P., van der Leest C., van der Spek P., Foekens J.A., Hoogsteden H.C. (2010). Gene expression-based classification of non-small cell lung carcinomas and survival prediction. PLoS ONE.

[26] Bhattacharjee A., Richards W.G., Staunton J., Li C., Monti S., Vasa P., Ladd C., Beheshti J., Bueno R., Gillette M. (2001). Classification of human lung carcinomas by mRNA expression profiling reveals distinct adenocarcinoma subclasses. Proc. Natl. Acad. Sci. USA.

[27] Faust P.L., Kovacs W.J. (2014). Cholesterol biosynthesis and ER stress in peroxisome deficiency. Biochimie.

[28] Adams C.M., Reitz J., De Brabander J.K., Feramisco J.D., Li L., Brown M.S., Goldstein J.L. (2004). Cholesterol and 25-hydroxycholesterol inhibit activation of SREBPs by different mechanisms, both involving SCAP and Insigs. J. Biol. Chem..

[29] Cui J., Sun W., Hao X., Wei M., Su X., Zhang Y., Su L., Liu X. (2015). EHMT2 inhibitor BIX-01294 induces apoptosis through PMAIP1-USP9X-MCL1 axis in human bladder cancer cells. Cancer Cell Int..

[30] Kim Y., Kim Y.S., Kim D.E., Lee J.S., Song J.H., Kim H.G., Cho D.H., Jeong S.Y., Jin D.H., Jang S.J. (2013). BIX-01294 induces autophagy-associated cell death via EHMT2/G9a dysfunction and intracellular reactive oxygen species production. Autophagy.

[31] Shimizu S., Kanaseki T., Mizushima N., Mizuta T., Arakawa-Kobayashi S., Thompson C.B., Tsujimoto Y. (2004). Role of Bcl-2 family proteins in a non-apoptotic programmed cell death dependent on autophagy genes. Nat. Cell Biol..

[32] Wesselborg S., Stork B. (2015). Autophagy signal transduction by ATG proteins: From hierarchies to networks. Cell. Mol. Life Sci..

[33] Otomo C., Metlagel Z., Takaesu G., Otomo T. (2013). Structure of the human ATG12~ATG5 conjugate required for LC3 lipidation in autophagy. Nat. Struct. Mol. Biol..

[34] An P.N.T., Shimaji K., Tanaka R., Yoshida H., Kimura H., Fukusaki E., Yamaguchi M. (2017). Epigenetic regulation of starvation-induced autophagy in Drosophila by histone methyltransferase G9a. Sci. Rep..

[35] Fan J.D., Lei P.J., Zheng J.Y., Wang X., Li S., Liu H., He Y.L., Wang Z.N., Wei G., Zhang X. (2015). The selective activation of p53 target genes regulated by SMYD2 in BIX-01294 induced autophagy-related cell death. PLoS ONE.

[36] Cao W., Ribeiro Rde O., Liu D., Saintigny P., Xia R., Xue Y., Lin R., Mao L., Ren H. (2012). EZH2 promotes malignant behaviors via cell cycle dysregulation and its mRNA level associates with prognosis of patient with non-small cell lung cancer. PLoS ONE.

[37] Seo Y.K., Jeon T.-I., Chong H.K., Beisinger J., Xie X., Osborne T.F. (2011). Genome-wide Localization of SREBP-2 in Hepatic Chromatin Predicts a Role in Autophagy. Cell Metab..

[38] Lim S.C., Parajuli K.R., Duong H.Q., Choi J.E., Han S.I. (2014). Cholesterol induces autophagic and apoptotic death in gastric carcinoma cells. Int. J. Oncol..

[39] Cheng J., Ohsaki Y., Tauchi-Sato K., Fujita A., Fujimoto T. (2006). Cholesterol depletion induces autophagy. Biochem. Biophys. Res. Commun..

[40] Cruz P.M.R., Mo H., McConathy W.J., Sabnis N., Lacko A.G. (2013). The role of cholesterol metabolism and cholesterol transport in carcinogenesis: A review of scientific findings, relevant to future cancer therapeutics. Front. Pharmacol..

[41] Sloan C.A., Chan E.T., Davidson J.M., Malladi V.S., Strattan J.S., Hitz B.C., Gabdank I., Narayanan A.K., Ho M., Lee B.T. (2016). ENCODE data at the ENCODE portal. Nucleic Acids Res..

[42] Dunham I., Kundaje A., Aldred S. (2012). An integrated encyclopedia of DNA elements in the human genome. Nature.

[43] Ashburner M., Ball C.A., Blake J.A., Botstein D., Butler H., Cherry J.M., Davis A.P., Dolinski K., Dwight S.S., Eppig J.T. (2000). Gene ontology: Tool for the unification of biology. The Gene Ontology Consortium. Nat. Genet..

[44] Croft D., O’Kelly G., Wu G., Haw R., Gillespie M., Matthews L., Caudy M., Garapati P., Gopinath G., Jassal B. (2011). Reactome: A database of reactions, pathways and biological processes. Nucleic Acids Res..

[45] Kuleshov M.V., Jones M.R., Rouillard A.D., Fernandez N.F., Duan Q., Wang Z., Koplev S., Jenkins S.L., Jagodnik K.M., Lachmann A. (2016). Enrichr: A comprehensive gene set enrichment analysis web server 2016 update. Nucleic Acids Res..

