# Supplementary material for: EHMT2 Inhibition Induces Cell Death in Human Non-Small Cell Lung Cancer by Altering the Cholesterol Biosynthesis Pathway
Source: Int J Mol Sci. 2020 Feb 3;21(3):1002. doi: 10.3390/ijms21031002 (PMC7037906; doi:10.3390/ijms21031002)
Supplement: Supplementary file 1 [file ijms-21-01002-s001.zip › ijms-687123-supplementary.pptx]

## Slide 1
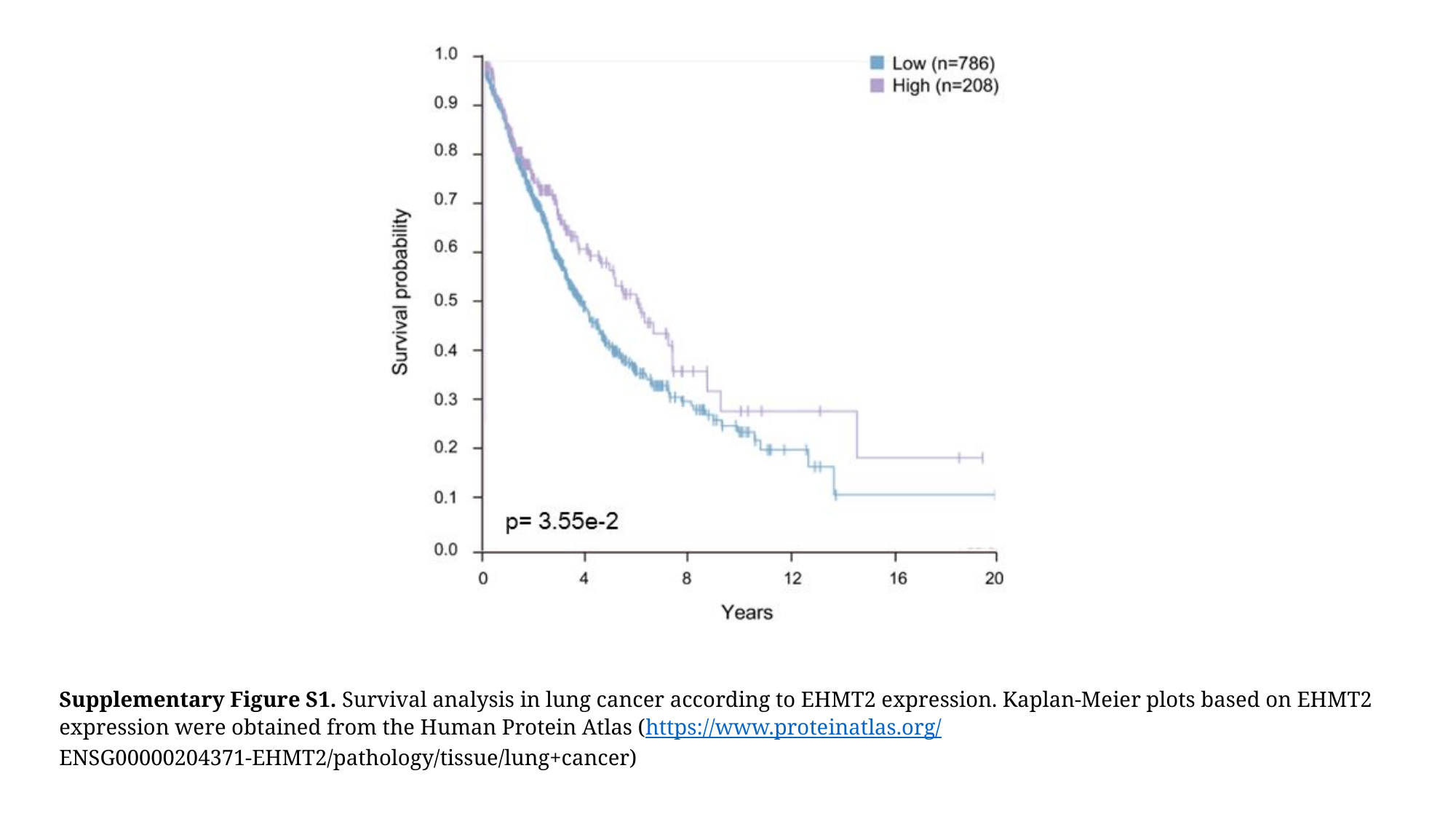

Supplementary Figure S1. Survival analysis in lung cancer according to EHMT2 expression. Kaplan-Meier plots based on EHMT2 expression were obtained from the Human Protein Atlas (https://www.proteinatlas.org/ ENSG00000204371-EHMT2/pathology/tissue/lung+cancer)

## Slide 2
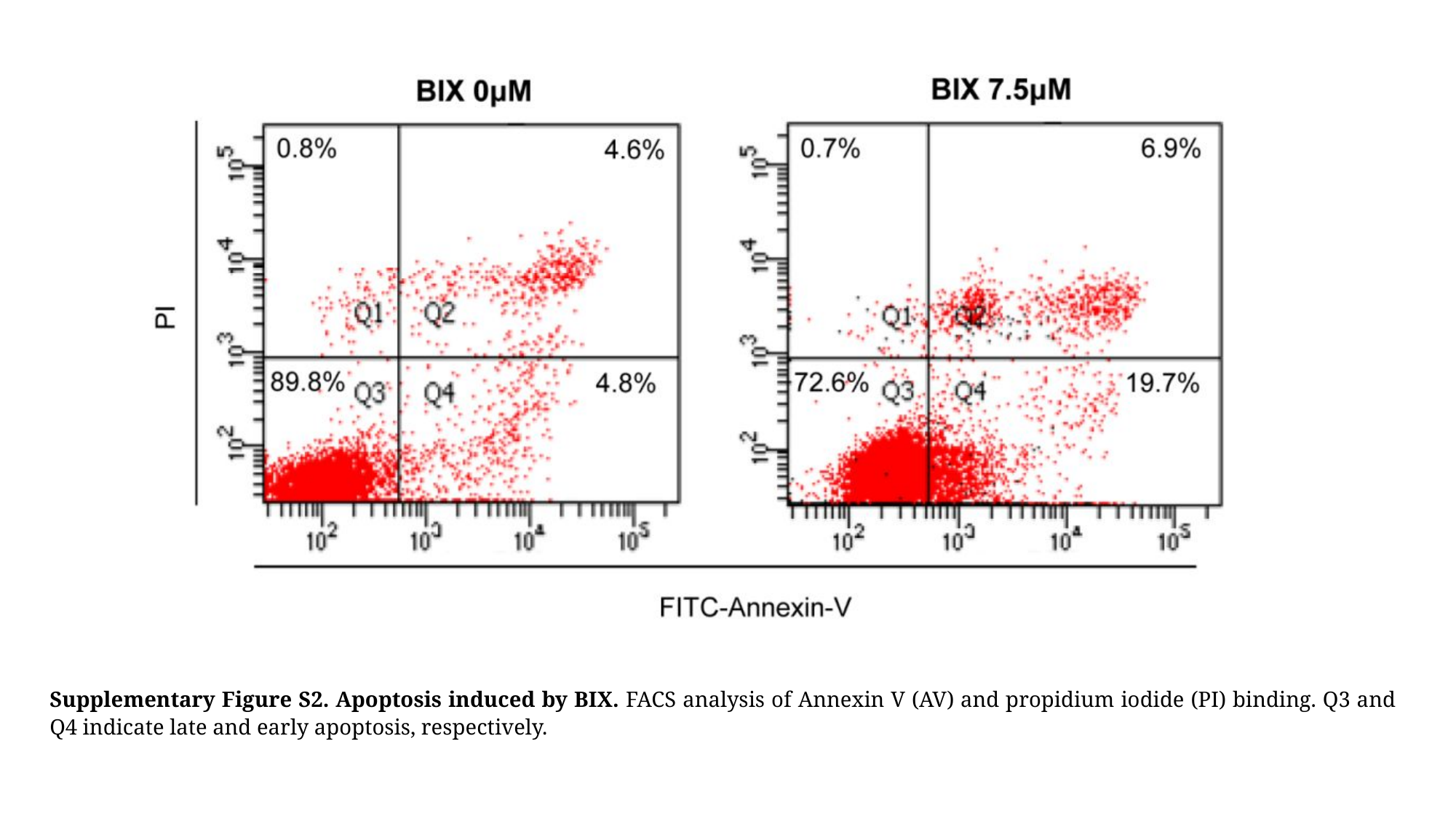

Supplementary Figure S2. Apoptosis induced by BIX. FACS analysis of Annexin V (AV) and propidium iodide (PI) binding. Q3 and Q4 indicate late and early apoptosis, respectively.
